# The Force–Velocity Profile for Jumping: What It Is and What It Is Not

**DOI:** 10.1249/MSS.0000000000003147

**Published:** 2023-03-18

**Authors:** MAARTEN F. BOBBERT, KOLBJØRN LINDBERG, THOMAS BJØRNSEN, PAUL SOLBERG, GØRAN PAULSEN

**Affiliations:** 1 Faculty of Behavioural and Movement Sciences, Department of Human Movement Sciences, Vrije Universiteit Amsterdam, Amsterdam Movement Sciences, THE NETHERLANDS; 2 Department of Sport Science and Physical Education, Faculty of Health and Sport Sciences, University of Agder, Kristiansand, NORWAY; 3 Norwegian Olympic Federation, Oslo, NORWAY; 4 Department of Physical Performance, Norwegian School of Sport Sciences, Oslo, NORWAY

**Keywords:** FORCE–VELOCITY RELATIONSHIP, LEG POWER, LOWER EXTREMITY, VALIDITY, SIMULATION MODEL

## Abstract

**Introduction:**

Force–velocity profiling has been proposed in the literature as a method to identify the overall mechanical characteristics of lower extremities. A force–velocity profile is obtained by plotting for jumps at different loads the effective work as a function of the average push-off velocity, fitting a straight line to the results, and extrapolating this line to find the theoretical maximum isometric force and unloaded shortening velocity. Here we investigated whether the force–velocity profile and its characteristics can be related to the intrinsic force–velocity relationship.

**Methods:**

We used simulation models of various complexity, ranging from a simple mass actuated by a linearly damped force to a planar musculoskeletal model comprising four segments and six muscle–tendon complexes. The intrinsic force–velocity relationship of each model was obtained by maximizing the effective work during isokinetic extension at different velocities.

**Results:**

Several observations were made. First, at the same average velocity, less effective work can be done during jumping than during isokinetic lower extremity extension at this velocity. Second, the intrinsic relationship is curved; fitting a straight line and extrapolating it seem arbitrary. Third, the maximal isometric force and the maximal velocity corresponding to the profile are not independent. Fourth, they both vary with inertial properties of the system.

**Conclusions:**

For these reasons, we concluded that the force–velocity profile is specific for the task and is just what it is: the relationship between effective work and an arbitrary estimate of average velocity; it does not represent the intrinsic force–velocity relationship of the lower extremities.

The vertical jumping ability of an athlete forms the basis for successful performance in numerous sports, such as volleyball and basketball. The theoretical maximum jump height that an athlete can reach is determined by the anatomical and physiological properties of that athlete’s musculoskeletal system, whereas the actual jump height reached also depends on the stimulation of each of the muscles as a function of time (1). If an athlete’s theoretical maximum jump height is to be increased, musculoskeletal properties need to be adapted by training, and the question becomes “Which factors are limiting jumping performance?” Samozino et al. (2) set out to answer this question by developing what they called a “theoretical integrative approach” to identify “the overall mechanical characteristics of lower extremities determining maximal jumping ability.”

Samozino et al. (2) identified three characteristics of the lower extremities determining maximal jumping ability: Δ*h*_PO_, ${\bar{F}}_{0}^{d}$, and ${\bar{v}}_{0}$; push-off distance Δ*h*_PO_ is the distance over which the lower extremity extends, and ${\bar{F}}_{0}^{d}$ and ${\bar{v}}_{0}$ are derived using a method called force–velocity profiling, which is explained in Figure 1. From a given initial squatted posture, a subject performs several maximum-effort jumps without countermovement, each with a different external load. For every jump, the vertical displacement of the center of mass in the airborne phase is determined (Fig. 1A) and used to calculate the gain in effective mechanical energy during the push-off. This gain in effective energy is determining jump height; it is the sum of the increase in potential energy and kinetic energy due to the vertical velocity of the center of mass during the push-off and equals the total change in potential energy from the start of the jump to the apex of the jump. The gain in effective energy is divided by Δ*h*_PO_ to yield what Samozino et al. (2) call the “average vertical force” (the outcome has the unit of force because J·m^−1^ = N·m·m^−1^ = N, but it is formally energy or muscle work averaged over distance). We will refer to this variable, expressed per kilogram of body mass, as ${\bar{F}}^{d}$. ${\bar{F}}^{d}$ is then combined with $\bar{v}$, the average vertical velocity of the center of mass during the push-off (Fig. 1B), to yield one point in the force–velocity profile (Fig. 1C). A straight line is fitted to the points of the different jumps, and this line is extrapolated to find ${\bar{F}}_{0}^{d}$, the intercept with the ${\bar{F}}^{d}$-axis, and ${\bar{v}}_{0}$, the intercept with the $\bar{v}$-axis. According to Samozino et al. (2), ${\bar{F}}_{0}^{d}$is the “theoretical maximal value of ${\bar{F}}^{d}$that lower extremities can produce during one extension at a theoretical null velocity,” and ${\bar{v}}_{0}$ is the “theoretical maximal value of $\bar{v}$ at which lower extremities can extend during one extension under the influence of muscles action in a theoretical unloaded condition.” ${\bar{F}}_{0}^{d}$ and ${\bar{v}}_{0}$, both depending on *h*_PO_, are claimed to be independent characteristics of the lower extremity; they are said to “characterize the mechanical limits of the entire neuromuscular function, encompassing individual muscle mechanical properties (e.g., intrinsic force–velocity and length–tension relationships, rate of force development), some morphological factors (e.g., cross-sectional area, fascicle length, pennation angle, tendon properties), and neural mechanisms (e.g., motor unit recruitment, firing frequency, motor unit synchronization, intermuscular coordination)” (6).

**FIGURE 1:**
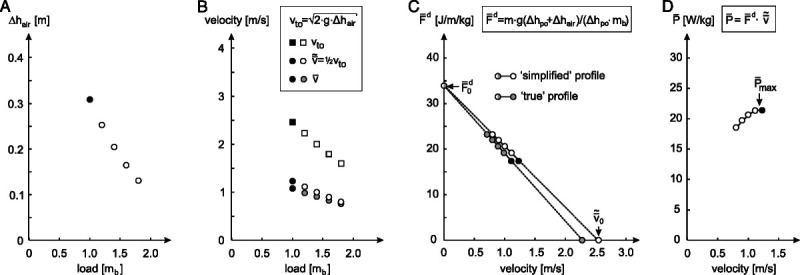
The method of force–velocity profiling for free squat jumps. From a standardized initial squatted posture, a subject performs five maximum height vertical squat jumps. One jump is unloaded (hence the load is body mass, *m_b_*), the other four are performed with extra load. For each jump, the height Δ*h*_air_ is determined, i.e., the vertical displacement of the center of mass in the airborne phase (A); from Δ*h*_air_, the takeoff velocity of the center of mass *v_to_* is calculated (B), as well as the total change in effective mechanical energy during the push-off (equal to the total increase in potential energy from the initial posture to the apex of the jump). The latter is divided by Δ*h*_PO_ to yield a variable that is referred to as “average vertical force” ${\bar{F}}^{d}$(it has the unit of force because J·m^−1^ = N·m·m^−1^ = N), which is expressed per kg body mass. ${\bar{F}}^{d}$ is then combined with $\bar{v}$, the average vertical velocity of the center of mass during the push-off, to yield one point in the force–velocity profile (C). A line is fitted to the points obtained from the different jumps, and this line is extrapolated to find ${\bar{F}}_{0}^{d}$ and ${\bar{v}}_{0}$, the intercepts with the ${\bar{F}}^{d}$-axis and the $\bar{v}$-axis, respectively. In the “simplified” profile, several approximations have been incorporated (2,3). For example, a fixed Δ*h*_PO_ (vertical displacement of the hips) is used regardless of the loading condition. In reality, the lower extremities may reach full extension during low-velocity jumps (at high loads), but they will not reach full extension during high-velocity unloaded jumps; this has to do with the dynamics of transforming segmental rotations into linear velocity of the center of mass (4). Also, Samozino et al. (2,3) do not use the actual mean velocity $\bar{v}$ but approximate it by $\tilde{\bar{v}}$, which is half the takeoff velocity *v_to_*; obviously, the two are only equal if the acceleration of the center of mass, and hence the ground reaction force, is constant (5). In the “true” profile, the actual vertical displacement of the hips during the push-off in each individual jump was used for Δ*h*_PO_, and the true $\bar{v}$ of the center of mass was used. Samozino et al. (2) propose to multiply ${\bar{F}}^{d}$and $\bar{v}$ to obtain “mean power output” $\bar{P}$, which has a maximum value of ${\bar{F}}_{0}^{d}\;{\bar{v}}_{0}/4$ (D). Symbols corresponding to the unloaded jump have been filled for convenience in reading the graphs. *m* is total mass (i.e., body mass plus externally added mass), *g* is gravitational acceleration.

Multiplication of ${\bar{F}}^{d}$ by $\bar{v}$ gives a variable with the unit of power, and plotting this variable against $\bar{v}$ yields a parabola (Fig. 1D), the apex of which is referred to as “maximum power” ${\bar{P}}_{\max}$ (6,7). It can be derived that ${\bar{P}}_{\max}$ is equal to $0.25\;{\bar{F}}_{0}\;{\bar{v}}_{0}$ (8). Because different combinations of ${\bar{F}}_{0}^{d}$ and ${\bar{v}}_{0}$ can yield the same ${\bar{P}}_{\max}$ (see, for example, Fig. 1 in [7]), the relationship between ${\bar{F}}_{0}^{d}$ and ${\bar{v}}_{0}$ is not uniquely defined by ${\bar{P}}_{\max}$ only. For that, ${\bar{P}}_{\max}$ needs to be combined with the slope of the line (− ${\bar{F}}_{0}^{d}/{\bar{v}}_{0}$), also referred to as the force–velocity profile (because ${\bar{F}}^{d}$is normalized for body mass, our definition corresponds to what in the literature is sometimes, but not consistently, called the “normalized force–velocity profile” [7,9]). Indeed, it was found that the variation in jump height among athletes could be better explained when, in addition to Δ*h*_PO_ and ${\bar{P}}_{\max}$, the profile was taken into account (7). Subsequently, it was proposed that for each individual, given his or her ${\bar{P}}_{\max}$ and Δ*h*_PO_, there is an optimal profile that maximizes unloaded jump height; the more the individual’s profile differs from the optimal one, the lower the performance in comparison with the one that could be reached with the same power capabilities (6,7). This opened the way to design specific training recommendations for individual athletes (6,10,11). Given the athlete’s ${\bar{P}}_{\max}$, an optimal slope is calculated (7,9), and based on the difference between the actual slope and the optimal slope, the so-called “force–velocity imbalance” (7), the athlete is classified as “force oriented” (the slope is more negative than optimal) or “velocity oriented” (the slope is less negative than optimal) (7,9). Along with this classification comes an individual training advice to improve jump height: a force-oriented athlete is recommended to participate in “speed-oriented training,” whereas a velocity-oriented athlete is recommended to participate in “strength-oriented training” (7,10,11). The idea is that such individualized training would reorient the athlete’s profile to the optimal profile; even in isolation, without a concomitant increase in ${\bar{P}}_{\max}$, such a reorientation would lead to an improvement in jumping performance (6,7,9,11).

The approach proposed by Samozino et al. (7,9) is popular in the field because it can easily be applied (3); in principle, a minimum of two jumps at different loads need to be executed by the athlete from a standardized initial posture (12–14), and the crucial variable jump height can be calculated from flight time (15), which can be determined using equipment as simple as a contact mat (16) or nowadays a smartphone (17). Although some investigators have reported amazing success of the approach of individualizing the training of athletes based on their force–velocity profiles (10,11), other authors failed to replicate the success (18–21) and have raised questions concerning the validity of the theoretical framework developed by Samozino et al. (2).

It needs no argument that the force–velocity relationship is an important limiting factor in a ballistic task like vertical jumping (22). The relationship is attributed to the coupling and decoupling of crossbridges between actin and myosin filaments (23–25), and it has an inherently hyperbolic shape at the level of single muscle fibers (e.g., [26–28]) and whole muscles (e.g., [29–31]). The relationship is formally determined by manipulating one variable and measuring the other. Hence, it can be determined by having a muscle perform isokinetic contractions at different velocities and measuring the maximal force that the muscle can produce at each velocity (e.g., [32–34]). Alternatively, it can be determined by having a muscle perform isotonic contractions at different constant forces and measuring the maximal shortening velocity that the muscle can reach at each force (e.g., [29]). However, this is not what is done in the force–velocity profiling approach; the load during the jumps is manipulated, but neither the force nor the velocity are controlled; both ${\bar{F}}^{d}$and $\bar{v}$ are calculated *post hoc* from the outcome of each jump. Given that the intrinsic force–velocity relationship of muscle is hyperbolic, why would fitting a straight line relationship to combinations of ${\bar{F}}^{d}$ and $\bar{v}$ obtained in jumps, and extrapolating this line to intersections with the axes, would yield variables that, according to Samozino et al. (2, p. 16, paragraph 4.2), “… do not only correspond to intrinsic muscle properties, but are the resultant of all the biological features affecting the maximal force that can be developed during lower extremities extension ( ${\bar{F}}_{0}^{d}$) and the maximal extension velocity ( ${\bar{v}}_{0}$)?”

In the present study, we revisited the “theoretical integrative approach” of force–velocity profiling for jumping. For this purpose, we used simulation models of various complexity. Our fundamental question was whether the force–velocity profile and its characteristics could be related to the intrinsic force–velocity relationship of the models.

## METHODS

In Matlab (MATLAB R2011b; The MathWorks Inc., Natick, MA) we developed simulation models of various complexity (35) and made them perform vertical “jumps” and isokinetic contractions. Each model consisted of a set of differential equations that were numerically solved after turning on the actuator(s) from an initial equilibrium situation. All models had the same total body mass (82 kg) and the same maximum push-off distance (0.4 m). Jump heights were calculated for unloaded jumps and for jumps with added loads ranging from 0 to 0.8 times body mass. From the resulting jumps, we determined the force–velocity profile using the equations proposed by Samozino et al. (2,3). Below, we will present each model, starting with the simplest model and ending with a full planar model of the human musculoskeletal system. The models were as follows:

### Model A1: a mass projected by a linearly damped force

**FIGURE 2:**
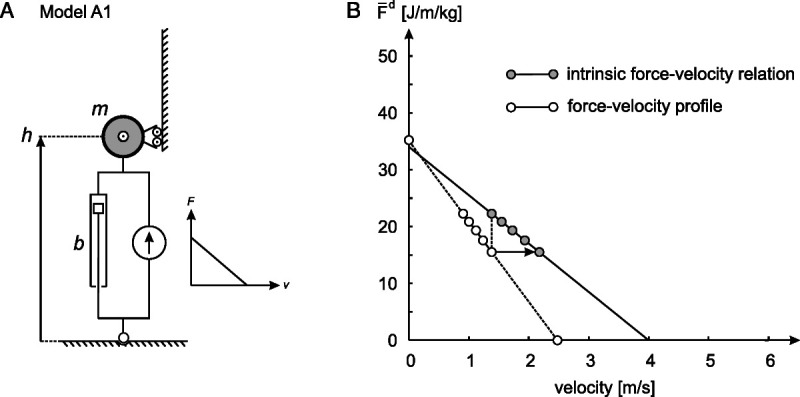
Model A1 and its force–velocity profile. A, Model A1 consists of a mass projected by a linearly damped force (inset). *m*, total mass (82 kg); *b*, coefficient of linear damping; *h*, height of the center of mass; *F*, force of the actuator; *v*, velocity of the actuator. The intrinsic force–velocity relationship of the actuator was *F* = *F*_0_ − *b* *v*, where isometric force *F*_0_ was 3400 N and *b* was 700 N·s·m^−1^. B, Force–velocity profile of the model obtained by plotting effective work per unit push-off distance ( ${\bar{F}}^{d}$) as a function of half the takeoff velocity (see Fig. 1). The force–velocity profile differs from the intrinsic relationship of the model. The latter is obtained by having the model extend isokinetically at different velocities. During the unloaded jump, less effective work is produced than during an isokinetic extension at the same average velocity (*vertical dashed line*). The same effective work as during the unloaded jump can be produced during an isokinetic extension at a higher velocity (*solid arrow*).

Model A1 (Fig. 2A) was an actuator consisting of an ideal source of force in parallel with a damper, which vertically projects a mass over a fixed push-off distance.

### Model A2: a mass projected by a nonlinearly damped force

Model A2 (Fig. 4A) was similar to model A1, but it had an intrinsic force–velocity relationship similar to the hyperbolic relationship proposed by Hill (30).

### Model B1: a mass projected by two massless segments driven by a nonlinearly damped actuator

Model B1 (Fig. 5A) consisted of a two-segment “lower extremity” with a “knee joint” driven by an actuator with a hyperbolic force–velocity relationship. Alls the mass was concentrated in the “hip joint.”

### Model B2: a mass projected by two inertial segments driven by a nonlinearly damped actuator

Model B2 (Fig. 6A) had the same properties as model B1 with one exception: its mass was no longer concentrated at the hip joint; rather, the segments had approximately the same inertial properties as those in a human body (36).

### Model C: four segments actuated by six Hill-type muscle–tendon complexes

Model C (Fig. 7A) comprised four body segments, actuated by six lumped muscle–tendon complexes of the human lower extremity. Each muscle–tendon complex was represented by a Hill-type unit, comprising a contractile element, a series elastic element, and a parallel elastic element. Forces of the elastic elements quadratically increased with elongation, whereas force of the contractile element depended on length and velocity of the contractile element, and active state (37). Active state, in turn, dynamically depended on muscle stimulation over time (STIM(*t*)). Initial STIM levels were set such that the model was in equilibrium in the starting position. During the jump, STIM of each muscle–tendon complex was allowed to change from the initial level to the maximum level of 1 at a rate of 5·s^−1^ (38), and this increase started at a STIM onset time. For a vertical jump, the combination of STIM onset times that maximized the height achieved by the center of mass was found using a genetic algorithm (39). For an isokinetic lower extremity extension, the toes were moved away from the hip at a constant velocity, and the combination of STIM onset times that maximized the work done on the toes was found.

### RESULTS

#### Model A1: a mass projected by a linearly damped force

Model A1 (Fig. 2A) was the simplest model we could think of to produce a force-velocity profile. It is a useful model because its intrinsic force–velocity relationship is identical to the intrinsic force–velocity relationship of the actuator (Fig. 2B). In the unloaded situation, the total mass equals body mass. Adding extra mass causes a reduction of the velocity reached at each given lower extremity length, and at that lower velocity, more force can be produced by the actuator. Hence, more work will be produced over the push-off, and we can generate the force–velocity profile of the system by varying the mass and applying the equations proposed by Samozino et al. (2) to the resulting jump outcome (see Fig. 1). The force–velocity profile of model A1 is shown in Figure 2B. What exactly does the profile represent? How is it related to the slope of the intrinsic force–velocity relationship? Because the vertical force on the mass is equal to the actuator force, and the actuator force is directly determined by the vertical velocity of the mass, one would hope that the slope of the relationship for the whole system is equal to the damping coefficient of the actuator. However, the relation between ${\bar{F}}^{d}$and $\bar{v}$ differs from the intrinsic force–velocity relationship; the latter declines less steeply and has a substantially higher extrapolated ${\bar{v}}_{0}$ (Fig. 2B). The relation between ${\bar{F}}^{d}$ and $\bar{v}$ would have matched the intrinsic relationship if ${\bar{F}}^{d}$ had been plotted as a function of 0.788 times the takeoff velocity rather than 0.5 times the takeoff velocity. The factor 0.788 was obtained *post hoc* from the outcome of the simulations.

**FIGURE 3:**
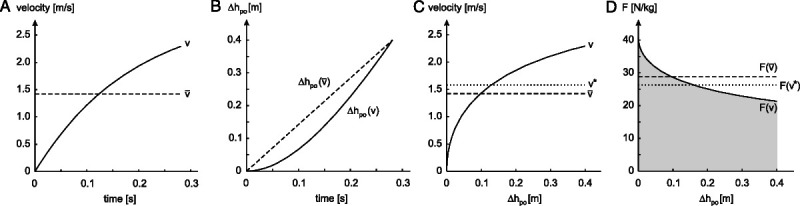
Simulation results for single lower extremity extensions of model A1. A, Time history of vertical velocity during the push-off for an unloaded jump (*v*) and for an isokinetic contraction at the average speed ( $\bar{v}$). Note that we calculated the true $\bar{v}$ for the jump, which was 1.42 m·s^−1^; approximating $\bar{v}$ by taking half the takeoff velocity in the jump (2,3) would have yielded a value of only 1.15 m·s^−1^ in this case. B, Vertical displacement during the push-off (Δ*h*_PO_) for corresponding lower extremity extensions. C, Velocity plotted as a function of Δ*h*_PO_. D. Vertical force (*F*) plotted as a function of Δ*h*_PO_. During an isokinetic contraction at the average velocity of the jump ( $\bar{v}$), more effective work is done (area below *F* ( $\bar{v}$)) than during the jump (*gray area*). At isokinetic velocity v* (higher than $\bar{v}$, see C), the same amount of work is done as during the jump.

Suppose that we wanted to determine the intrinsic relationship from measurements on the system as a whole, what would we have to make the system do? Well, the answer is simple: we could make the model contract isokinetically at different velocities; at each velocity, we would determine the effective work produced and divide it by the distance over which the model exerted force. This yields exactly the intrinsic relationship. In Fig. 2B, we have shown for each of the data points used to construct the force–velocity profile, the corresponding combination of ${\bar{F}}^{d}$ and the isokinetic velocity at which the same amount of effective work is produced. This isokinetic velocity is higher than $\bar{v}$. This can be understood as follows. Remember that the effective work is the integral of ${\bar{F}}^{d}$over push-off distance Δ*h*_PO_ and, hence, equals the integral of the vertical force *F* over Δ*h*_PO_; this integral is shown in Figure 3D for the unloaded jump (at the vertical line in Fig. 2B). Because the velocity increases during the push-off (Fig. 3A), the distance traveled at velocities higher than $\bar{v}$ is greater than the distance traveled at velocities lower than $\bar{v}$ (Fig. 3C). Consequently, less effective work is done during the jump than during an isokinetic contraction at $\bar{v}$ (Fig. 2B, dashed vertical line). The isokinetic velocity v* at which the amount of effective work produced equals the amount of effective work produced during the free jump (Fig. 2B, solid arrow) is higher than $\bar{v}$ (Fig. 3C and D). In this simple model, where the intrinsic force–velocity relationship of the model is linear, v* equals the velocity during the push-off averaged over push-off distance. The results shown in Fig. 3 already make clear that a force–velocity profile is restricted to the specific velocity–time history and hence is specific to the task.

In sum, although the effective work is a true outcome variable of the jump, expressing it as a function of half the takeoff velocity is an arbitrary choice; half the takeoff velocity is not at all the speed that determines the effective work. This also means that the maximal “power” calculated from the force–velocity profile (Fig. 1D) is only a relevant variable within the framework of the profile and is not a valid measure for the maximal power produced during jumping.

#### Model A2: a mass projected by a nonlinearly damped force

**FIGURE 4:**
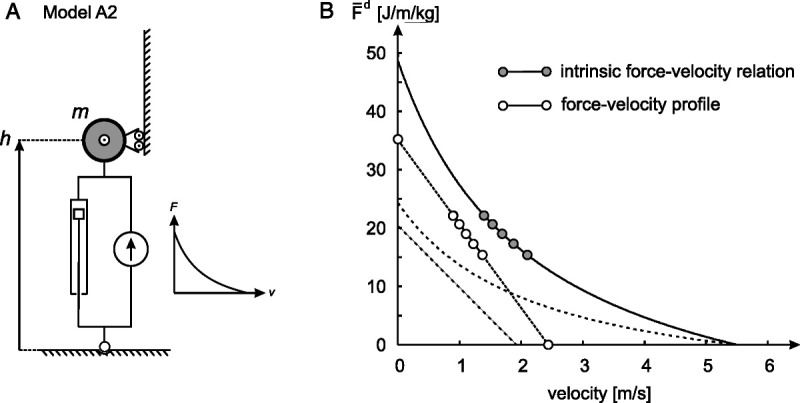
Model A2 and its force–velocity profile. A, Model A2 consists of a mass projected by a nonlinearly damped force (inset). Abbreviations as in Figure 2. The intrinsic force–velocity relationship of the actuator was (*F* + *a*) (*v* + *b*) = *b*(*F*_0_ + *a*), where *F*_0_ is isometric force (4000 N), and *a* (1600 N) and *b* (2.2 m·s^−1^) are constants. B, Force–velocity profile of the model obtained by plotting effective work per unit push-off distance ( ${\bar{F}}^{d}$) as a function of half the takeoff velocity (see Fig. 1). The force–velocity profile differs from the intrinsic relationship of the model. The latter is obtained by having the model extend isokinetically at different velocities. If the maximal force of the intrinsic relationship is reduced but the maximal velocity is kept constant, the maximal velocity of the force–velocity profile is reduced (*lowermost dashed lines*).

Model A2 (Fig. 4A) was similar to model A1 but had a hyperbolic force–velocity relationship rather than a linear one. The relationship is shown in Figure 4B, together with the force–velocity profile. For the five jumps that were used to construct the profile, we took the effective work and determined the isokinetic velocity v* at which the same amount of effective work could be produced; these are the data points shown on the intrinsic force–velocity relationship in Figure 4B. Because the intrinsic force–velocity relationship of the model is nonlinear, v* is no longer the average of velocity over distance, as it was in model A1. Just as the data points of the force–velocity profile, the v* data points seem to fall on a straight line that can be extrapolated to calculate values for ${\bar{F}}_{0}^{d}$ and ${\bar{v}}_{0}$, but this is a meaningless operation because the intrinsic relationship is nonlinear. The true ${\bar{F}}_{0}^{d}$ and ${\bar{v}}_{0}$ can only be found by fitting a hyperbola to the data points, which can successfully be done if we know the underlying relationship, but is doomed to fail if we need to determine and extrapolate the relationship using noisy experimental data. Because the straight line is fitted to only a small section of the nonlinear relationship, ${\bar{F}}_{0}^{d}$ and ${\bar{v}}_{0}$ are no longer independent; if we reduce the force of the intrinsic relationship and keep the maximal velocity constant, ${\bar{v}}_{0}$ decreases where it should not (lowermost dashed lines in Fig. 4B).

#### Model B1: a mass projected by two massless segments driven by a nonlinearly damped actuator

**FIGURE 5:**
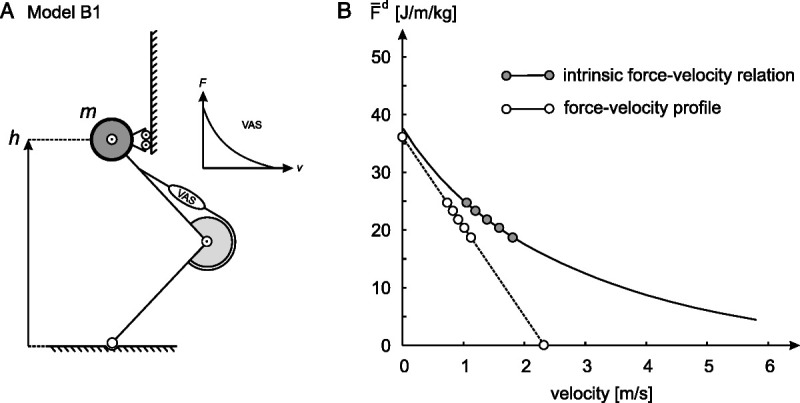
Model B1 and its force–velocity profile. A, Model B1 consists of a mass projected by two massless segments driven by a nonlinearly damped actuator (inset), labeled VAS (“vasti”). Abbreviations as in Figure 2. The length of each of the segments was 0.5 m. The intrinsic force–velocity relationship of VAS was (*F* + *a*) (*v* + *b*) = *b*(*F*_0_ + *a*), where *F* is force, *v* is shortening velocity, *F*_0_ is isometric force (15000 N), and *a* (6000 N) and *b* (0.72 m·s^−1^) are constants. The moment arm of VAS was 0.05 m. B, Force–velocity profile of the model obtained by plotting effective work per unit push-off distance ( ${\bar{F}}^{d}$) as a function of half the takeoff velocity (see Fig. 1). The force–velocity profile differs from the intrinsic relationship of the model. The latter is obtained by having the model extend isokinetically at different velocities.

In models A1 and A2, the intrinsic force–velocity relationship of the model was identical to the force–velocity relationship of the actuator. This was no longer the case in two-segment model B1 (Fig. 5A), in which the nonlinear geometrical relationship between actuator length and lower extremity length is now involved in the translation from actuator length changes to lower extremity length changes and from the actuator force to the force produced on the ground and on the mass (40). The intrinsic force–velocity relationship of the model, which we obtained by calculating the effective work done in isokinetic contractions at different velocities, is not represented by the force–velocity profile (Fig. 5B).

#### Model B2: a mass projected by two inertial segments driven by a nonlinearly damped actuator

**FIGURE 6:**
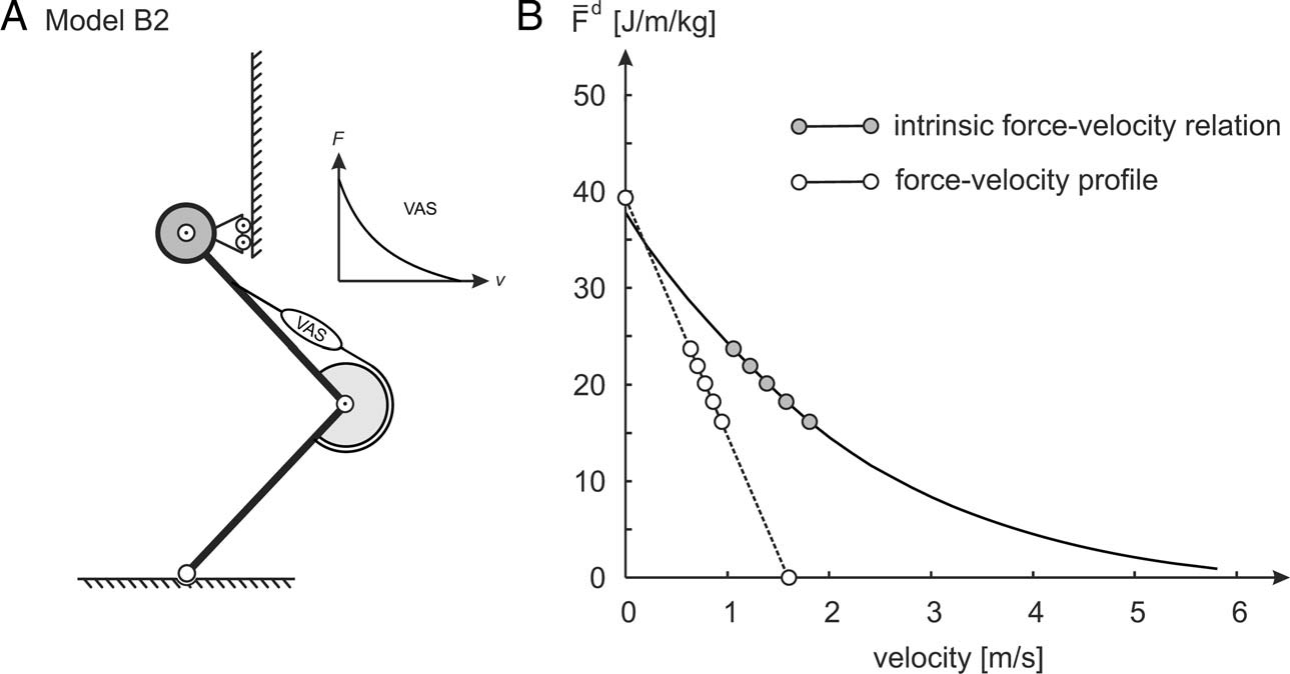
Model B2 and its force–velocity profile. Model B2 (A) is similar to model A2 (Fig. 5); the actuator properties and total mass are the same, but the mass is now distributed and the segments have inertial properties. This affects both the intrinsic relationship and the force–velocity profile (B). Skeletal model details: length of each of the segments, 0.5 m; mass of upper and lower leg segments, 16 and 7 kg, respectively; moments of inertia of upper and lower leg segments, 1.13 and 0.15 kg·m^−2^, respectively.

Model B2 (Fig. 6A) has the same actuator properties as model B1, but the mass is distributed and the segments have inertia. As a result, part of the energy produced by the actuator ends up as rotational energy of the segments in model B2. Hence, less energy is available to project the mass against gravity, and lower velocities are reached in a jump at a given load compared with model B1. This explains why in model B2, compared with model B1, ${\bar{F}}^{d}$ fell off more quickly with $\bar{v}$, ${\bar{v}}_{0}$ decreased, and ${\bar{F}}_{0}^{d}$ increased (cf. Figs. 5B and 6B). In particular, the change in ${\bar{F}}_{0}^{d}$ is unexpected because according to Samozino et al. (2) it is the “… theoretical maximal value of ${\bar{F}}^{d}$ that lower extremities can produce during one extension at a theoretical null velocity.” During an infinitesimally slow contraction, the inertial properties of the model should not matter; this is true for the intercept of the intrinsic relationship but turns out to be false for ${\bar{F}}_{0}^{d}$ of the profile.

#### Model C: four segments actuated by six Hill-type muscle–tendon complexes

**FIGURE 7:**
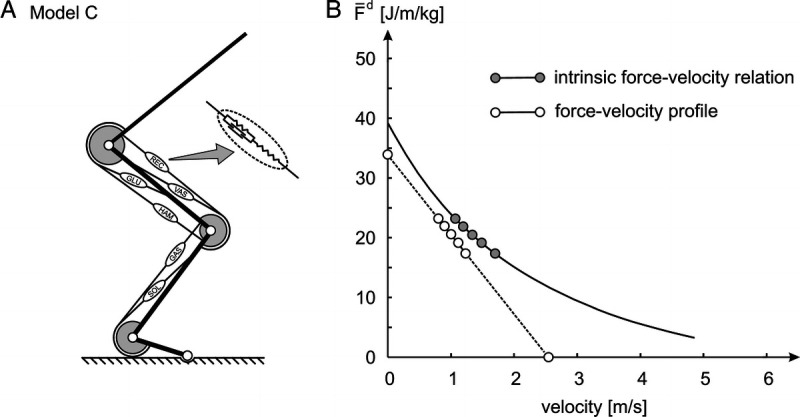
Model C and its force–velocity profile. A, Model C comprised four body segments actuated by six major muscle–tendon complexes of the human lower extremity. Each muscle–tendon complex was represented by a Hill-type unit, comprising a contractile element, a series elastic element and a parallel elastic element (inset). The only input was the stimulation of each of the units over time, which was optimized to maximize the effective work. B, Force–velocity profile of the model obtained by plotting effective work per unit push-off distance ( ${\bar{F}}^{d}$) as a function of half the takeoff velocity (see Fig. 1). The force–velocity profile differs from the intrinsic relationship of the model, obtained by having the model extend isokinetically at different velocities. SOL, m. soleus; GAS, m. gastrocnemius; VAS, mm. vasti; REC, m. rectus femoris; GLU, m. gluteus maximus; HAM, hamstrings.

The most complex model we used in this study comprised four body segments, actuated by six lumped muscle–tendon complexes of the human lower extremity (Fig. 7A). It has been shown elsewhere that this model is capable of successfully reproducing human vertical jumps (38,41), and the model was actually used to create Figure 1. Figure 7B shows the force–velocity profile of the model as well as the relationship obtained using isokinetic lower extremity extensions. If we define the latter relationship as the force–velocity relationship of the lower extremities, this relationship is not in any way represented by the profile or its characteristics ${\bar{F}}_{0}^{d}$ and ${\bar{v}}_{0}$.

## DISCUSSION

Force–velocity profiling as explained in Figure 1 has been proposed in the literature as a method to identify the “overall mechanical characteristics of lower extremities determining maximal jumping ability” (2). Furthermore, it is used to give individualized training recommendations to athletes (10,11). In the present study, we investigated whether the force–velocity profile and its characteristics could be related to the intrinsic force–velocity relationship of the lower extremity as a whole. For that purpose, we used simulation models of various complexity. The intrinsic relationship of each model was defined as the relationship obtained by having the model perform isokinetic “lower extremity” extensions at various velocities and determining the maximal amount of effective work that could be produced at each velocity. This definition was based on our most simple model: a mass projected by a linearly damped force (model A1; Fig. 2); how else could we define the intrinsic relationship of that model? We found that the intrinsic relationships of our models were not represented by the force–velocity profiles or their characteristics ${\bar{F}}_{0}^{d}$ and ${\bar{v}}_{0}$. Below, we will discuss the most important reasons, the question whether force–velocity profiles of different tasks can be compared, and the question whether it is problematic that the force–velocity profile does not reflect the intrinsic force–velocity relationship.

The most important reasons why the intrinsic force–velocity relationship is not represented by the force–velocity profile and its characteristics ${\bar{F}}_{0}^{d}$ and ${\bar{v}}_{0}$ are the following. First, the amount of effective work produced during lower extremity extension does not depend on the average velocity but on the time history of the velocity: at the same average velocity, less effective work can be done during jumping than during isokinetic lower extremity extension at this velocity (Fig. 2B, vertical dashed line). The results shown in Figure 3 make clear that the effective work during a lower extremity extension depends on the specific velocity–time history, which depends on the task. Hence, the force–velocity profile is also specific to the task. Although the effective work in jumping can be determined unambiguously, the choice of Samozino et al. (2) to express it as a function of half the takeoff velocity—as a proxy for the average velocity—is arbitrary. This is already clear for model A1; as we pointed out in the Results section; if ${\bar{F}}^{d}$ had been expressed as 0.788 times rather than 0.5 times the takeoff velocity, the force–velocity profile would have matched the intrinsic relationship in Figure 2B. However, the factor is unknown beforehand because it depends on the dynamics of the lower extremity extension task and also on the intrinsic relationship itself. Even worse, if the relationship is nonlinear, as in model A2, the factor is different for each jump (Fig. 4B). Second, the combinations of ${\bar{F}}^{d}$ and $\bar{v}$ that are achieved over a realistic range of jump loadings cover only a small part of the force–velocity relationship. The combinations can be fitted with a straight line, but they may just as well lie on a curve (Figs. 4–7). Obviously, the extrapolated line yields other intersections with the axes than the extrapolated curve. Because the experimental data are insufficient to decide whether a line or a curve should be fitted, extrapolations are bound to be invalid. This is true even for ${\bar{F}}_{0}^{d}$ (e.g., Fig. 4B). Third, and related to the fitting of a line to a section of the nonlinear relationship, ${\bar{F}}_{0}^{d}$ and ${\bar{v}}_{0}$ are not independent characteristics, in contrast to what was claimed in the literature (2,6): if we reduce the force of the intrinsic relationship and keep the maximal velocity constant, ${\bar{v}}_{0}$ decreases (Fig. 4B). Fourth, at a given intrinsic relationship, ${\bar{F}}_{0}^{d}$ and ${\bar{v}}_{0}$ of a segmented model depend on the inertial properties of the system (cf. Figs. 5B and 6B). For these reasons, the force–velocity profile for human jumping (Fig. 7B) does not represent the intrinsic force–velocity relationship of the lower extremities. Rather, it is just what it is: the empirical relationship between effective work (divided by a constant push-off distance) and half the takeoff velocity in jumps at different loads.

With their “theoretical integrative approach,” Samozino et al. (2,6) intended to characterize the “dynamic mechanical capabilities of the neuromuscular system during a lower limb extension.” The question may be raised as to how general this characterization is. A force–velocity profile can be determined for lower extremity extension during jumping (Figs. 1 and 6), but also for lower extremity extension on a ballistic dynamometer (6), a pneumatic dynamometer (42), or an isotonic dynamometer (43–45). The effective work produced during these different lower extremity extensions can be determined unambiguously and can be divided by push-off distance to obtain ${\bar{F}}^{d}$. However, what should be put on the velocity axis? We have established in the present study that average velocity $\bar{v}$ is not a representative variable because the force–velocity profile depends on the dynamics of the task (Fig. 3). Apart from that, there is a difference in the range of motion in the hip joints between jumping and lower extremity extensions on a dynamometer; in jumping, these joints can fully extend, which allows the hip extensor muscles to produce work over their full range, whereas on a leg press dynamometer the extension of the hip joints is limited because the trunk is fixed. Hence, when comparing force–velocity profiles for different tasks (e.g., [46,47]), we may expect them to be dissimilar.

Having established that the force–velocity profile for a task like jumping does not represent the intrinsic force–velocity relationship of the lower extremity, the question may be raised whether this is problematic. One of the applications of the approach is in designing training programs based on the force–velocity imbalance, i.e. the difference between the actual and optimal force–velocity profiles of an individual (7,10,11). To improve their jump height, force-oriented athletes are recommended to participate in speed-oriented training, whereas velocity-oriented athletes are recommended to participate in strength-oriented training. Regardless of the debate as to whether this individualized training approach works better than traditional approaches (10,18–21), the question may be raised whether the force–velocity profile itself is needed to classify athletes. Neither the effective work nor the average velocity are independently manipulated, and the use of average velocity on the horizontal axis seems arbitrary, as we have argued in this article. When it comes to improving jumping ability, it would be more straightforward to put on the horizontal axis the truly independently manipulated load and on the vertical axis the resulting jump height (Fig. 1A) or the takeoff velocity (Fig. 1B). An athlete who is relatively strong but does not jump high may be classified as force oriented, and an athlete who is relatively weak but jumps high may be classified as velocity oriented. From a training perspective, the important question is what underlies these “orientations.” The intrinsic force–velocity relationship will surely play a role but is unlikely to be the only factor.

## CONCLUSIONS

We conclude from the findings in this study that the force–velocity profile for jumping does not represent the intrinsic force–velocity relationship of the lower extremities and does not have added value over plotting jump height or takeoff velocity as a function of the truly independently manipulated load.
